# Modeled microgravity alters lipopolysaccharide and outer membrane vesicle production of the beneficial symbiont *Vibrio fischeri*

**DOI:** 10.1038/s41526-021-00138-8

**Published:** 2021-03-08

**Authors:** Madeline M. Vroom, Yaneli Rodriguez-Ocasio, Jonathan B. Lynch, Edward G. Ruby, Jamie S. Foster

**Affiliations:** 1 Department of Microbiology and Cell Science, Space Life Science Lab, University of Florida, Merritt Island, FL USA; 2 Pacific Biosciences Research Center, Kewalo Marine Laboratory, University of Hawaiʻi at Manoa, Honolulu, HI USA; 3 Department of Integrative Biology and Physiology, University of California, Los Angeles, CA USA

**Keywords:** Microbiology, Zoology

## Abstract

Reduced gravity, or microgravity, can have a pronounced impact on the physiology of animals, but the effects on their associated microbiomes are not well understood. Here, the impact of modeled microgravity on the shedding of Gram-negative lipopolysaccharides (LPS) by the symbiotic bacterium *Vibrio fischeri* was examined using high-aspect ratio vessels. LPS from *V. fischeri* is known to induce developmental apoptosis within its symbiotic tissues, which is accelerated under modeled microgravity conditions. In this study, we provide evidence that exposure to modeled microgravity increases the amount of LPS released by the bacterial symbiont in vitro. The higher rates of shedding under modeled microgravity conditions are associated with increased production of outer-membrane vesicles (OMV), which has been previously correlated to flagellar motility. Mutants of *V. fischeri* defective in the production and rotation of their flagella show significant decreases in LPS shedding in all treatments, but levels of LPS are higher under modeled microgravity despite loss of motility. Modeled microgravity also appears to affect the outer-membrane integrity of *V. fischeri*, as cells incubated under modeled microgravity conditions are more susceptible to cell-membrane-disrupting agents. These results suggest that, like their animal hosts, the physiology of symbiotic microbes can be altered under microgravity-like conditions, which may have important implications for host health during spaceflight.

## Introduction

All animals form beneficial associations with microbes, and these symbioses, collectively called the microbiome, constitute a powerful determinant of development, health, and fitness^1,2^. Indeed, alterations in the composition of the microbiome, its secretion of bioactive metabolites, as well as the symbionts’ resultant with host systems (e.g., nervous, immune, endocrine) have been found to correlate with a variety of illnesses and disorders^2–4^. Understanding how mutualistic microbes adapt to stressful conditions within animal hosts, including humans, therefore, represents a crucial topic for microbiome research.

One environment that presents numerous challenges to host–microbe homeostasis is spaceflight^5^. In particular, the microgravity conditions (10^−6^ × g) that astronauts experience during spaceflight have been shown to disrupt the normal physiology of the human body, including the intestinal microbiome^6,7^. This dysbiotic response is characterized by a decline in gut bacteria predicted to be protective, including *Bifidobacterium* and *Lactobacillus* spp.; the increased abundance of opportunistic pathogens like *Escherichia coli, Enterobacteria* spp., and *Clostridia* spp.; as well as shifts in the relative proportions of dominant taxa from the Firmicutes and Bacteroidetes^6–12^.

In addition to measuring the relative abundance of various bacterial populations, many studies have also sought to determine the impact of the spaceflight on the physiology of those taxa. In this regard, microgravity and microgravity-analog conditions have been found to increase the resistance of opportunistic pathogens, such as *E. coli* and *Pseudomonas aeruginosa*, to acid, heat, osmotic, and oxidative stresses^13–15^. Cultivation in these conditions is also known to promote biofilm formation and enhance the virulence of certain pathogens, including *Salmonella enterica* serovar Typhimurium^16–19^ and *Serratia marcescens*^20^. Though much progress has been made in delineating how pathogenic microbes respond to this unique stressor, the larger discussion of how beneficial microbes respond to microgravity has only recently begun to be examined^21–27^.

One mutualistic association that has emerged as a tractable model by which to examine beneficial host–microbe interactions under modeled and natural microgravity conditions is the monospecific symbiosis between the Hawaiian bobtail squid, *Euprymna scolopes*, and its bioluminescent partner *Vibrio fischeri*^21,24,28^. Upon hatching, *V. fischeri* colonizes a specialized organ, known as the light organ, within the host animal and induces an irreversible remodeling of the symbiotic tissues^29^. The remodeling is marked by a pronounced apoptotic cell death event throughout the light organ’s superficial, ciliated epithelium^30,31^, a structure that is used to recruit symbiosis-competent *V. fischeri* from the environment and facilitate the onset of the association.

**Fig. 1:**
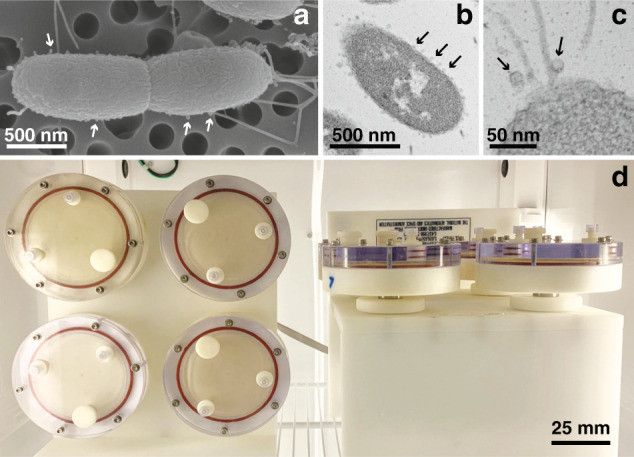
Overview of the beneficial microbe *Vibrio fischeri* morphology and experimental conditions. **a** Scanning electron micrograph of the wild-type *V. fischeri* depicting the presence of outer-membrane vesicles (OMVs) on the cell surface (arrows). **b** Transmission electron micrograph (TEM) of *V. fischeri* during exponential growth producing numerous OMVs during exponential-phase growth. **c** Higher magnification TEM visualizing OMVs associated with the bacteria flagella. **d** Rotary cell culture system with high-aspect ratio vessels containing *V. fischeri* cultures in the modeled microgravity (left) and gravity (right) control positions.

Two microbe-associated molecular pattern signals (MAMPs) that have been identified as the primary mediators of the *V. fischeri*-induced apoptotic event in *E. scolopes* are LPS and the peptidoglycan-derived monomer, tracheal cytotoxin (TCT)^32,33^. These two MAMPs work synergistically to induce apoptosis and regression of the light organ’s ciliated epithelium during the onset of symbiosis^33,34^. Early-stage apoptosis within the host tissues, however, is primarily attributed to LPS^32^, and most of the LPS released by *V. fischeri* is embedded in outer-membrane vesicles (OMVs) that are shed via rotation of the bacterium’s sheathed, polar, flagella (Fig. 1a–c)^35,36^. The rapid onset of MAMP-inducible phenotypes, combined with the binary nature of the light organ symbiosis, has rendered the squid-vibrio system a uniquely informative model for the study of animal-microbial mutualisms in microgravity^22^.

Several ground-based systems exist that enable microgravity-like conditions to be modeled, including high-aspect ratio vessels (HARVs). HARVs mimic the quiescent, low-shear, fluid conditions of spaceflight when rotated around a horizontal axis (Fig. 1d)^22,37^. Alternatively, rotation around a vertical axis is used to control for system-specific effects under normal Earth gravity conditions^22,38^. This low-shear modeled microgravity (LSMMG) environment has been used to successfully mimic microgravity for decades, and has been correlated to results obtained during actual spaceflight^13,23,37^.

Previous work with the squid-vibrio symbiosis using LSMMG has found that the normal symbiont-induced development of the light organ is altered by LSMMG^21,23,24,27^. Specifically, modeled microgravity accelerates both the onset and peak of apoptosis in both symbiotic and aposymbiotic, LPS-treated hatchlings^21^. Given the role of LPS in the normal bacteria-induced development of *E. scolopes*, these findings raised the possibility that LSMMG may alter MAMP-mediated channels of host–microbe communication. MAMPs are a crucial component of both beneficial and pathogenic interactions^39^; therefore, in this study, we examined the impact of LSMMG on the release of LPS and OMVs by *V. fischeri*, and assessed the overall physiology of the symbiont when cultivated under microgravity-like conditions. Examining the effects of modeled microgravity on beneficial microbes has the potential to both provide new insight on the underlying mechanisms of microgravity-induced dysbiosis and further our understanding of mutualistic host-microbe interactions during spaceflight.

## Results

### Shedding of *V. fischeri*-derived LPS increased during exponential growth under LSMMG conditions compared to gravity controls

To determine the potential impact of modeled microgravity on LPS release during growth, cultures of wild-type *V. fischeri* ES114 were grown in HARV reactors in the LSMMG and gravity positions (Fig. 1d) and monitored at 2–4-h intervals. The growth curves were characteristic of several microbes cultivated under LSMMG, including *V. fischeri*, exhibiting increased growth rate during exponential phase compared to gravity controls (Supplementary Fig. 1)^23,27,40^. At each growth interval, the *V. fischeri* cells were removed from the culture media and the level of exogenous LPS shed by the bacteria was measured and compared using two independent assays. First, the colorimetric ToxinSensor Chromogenic Limulus Amebocyte Lysate (LAL) endotoxin assay, which measures the biological reactivity of the lipid A moiety of LPS, was used to measure reactogenic LPS in the cell-free medium filtrate. The second method, the Purpald assay, is based on the periodate oxidation of sugars of the core regions of the LPS molecule^41^, and was used on the same, cell-free media filtrate to quantify the total amount of LPS. Although complementary, these two assays target different components of the LPS molecule^42^.

**Fig. 2:**
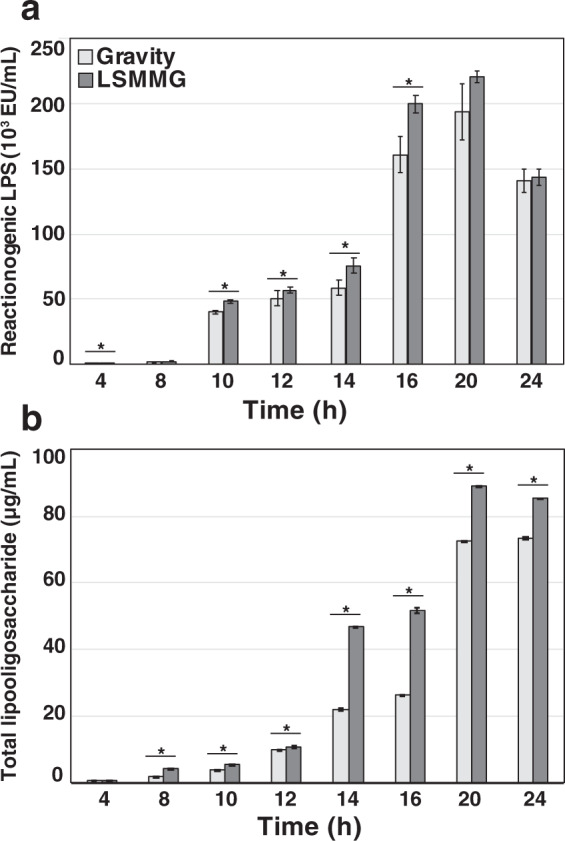
Lipopolysaccharide (LPS) shedding by *Vibrio fischeri* wild-type ES114 under low-shear modeled microgravity (LSMMG) and gravity conditions. **a** Reactogenic LPS as measured by the limulus amebocyte lysate endotoxin assay under gravity (light gray) and LSMMG (dark gray) conditions. **b** Total LPS levels as measured by the Purpald assay between LSMMG and gravity (G) conditions. Error bars indicate the standard error of the mean. Asterisks indicate significant differences between the data sets (*p* < 0.05; Mann–Whitney U test).

During exponential growth in the HARV reactors, *V. fischeri* cells released significantly more LPS under LSMMG conditions than gravity controls even after being normalized by optical density, an indicator of cell mass (Fig. 2a). Levels of reactogenic LPS in the surrounding media, as determined by the LAL endotoxin assay, showed a pronounced increase beginning 10 h post inoculation (Fig. 2a). The significantly higher levels of LPS released from the LSMMG-grown cells continued until ~18–20 h, after which no differences in LPS levels were observed between the two growth conditions. Measurement of total LPS by the Purpald assay showed a similar increase, with the concentrations of total LPS shedding in LSMMG cultivars compared to gravity controls higher at all tested time points between 8 and 24 h post inoculation (Fig. 2b).

### Increased LPS release during modeled microgravity is correlated, but not completely dependent on, cell motility

Previous work has shown that most of the LPS released by *V. fischeri* is embedded in OMVs that are derived from rotation of the outer-membrane-sheathed, polar flagella^35,36,43^. This result led us to determine whether LSMMG stimulates bacterial motility, which would ostensibly increase vesiculation and, as a corollary, the shedding of LPS. Interestingly, the motility of wild-type cells grown under either LSMMG or gravity conditions showed no statistical differences when examined using direct microscopic video analysis or soft-agar motility assays (Supplementary Fig. 2). In addition, we also compared the amount of LPS shedding by two motility-deficient *V. fischeri* mutants, *motB1* and *flhA*, grown under gravity and LSMMG conditions. Mutants defective in *motB1*, which encodes for a flagellar motor protein, do form flagella but are unable to rotate them, whereas *flhA* mutants are defective in the synthesis of flagella^44,45^.

**Fig. 3:**
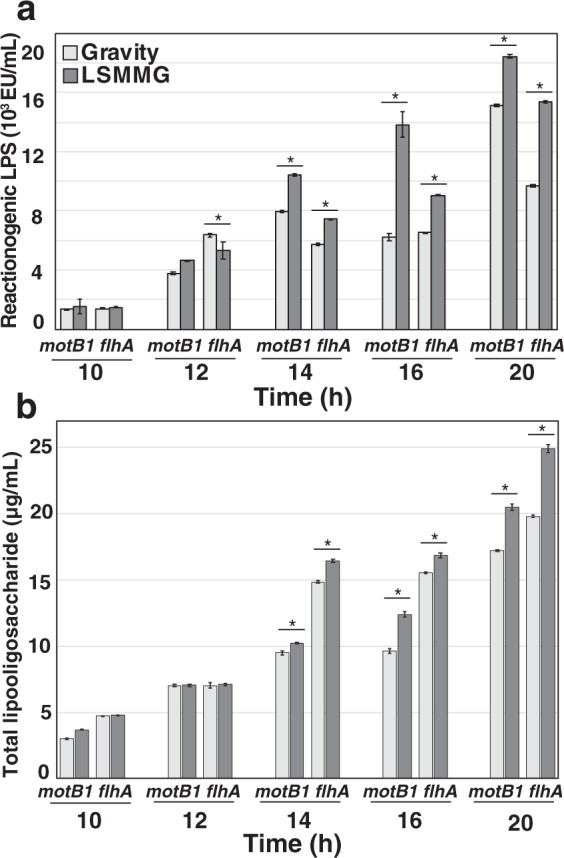
Lipopolysaccharide (LPS) shedding of *V. fischeri* motility mutants *motB1* and *fhlA* under low-shear modeled microgravity (LSMMG) conditions compared to gravity controls. **a** Reactionogenic LPS as measured by the limulus amebocyte lysate endotoxin assay under gravity (light gray) and LSMMG (dark gray) conditions. **b** Total LPS levels as measured by the Purpald assay between LSMMG and gravity (G) conditions. Error bars indicate the standard error of the mean. Asterisks indicate significant differences between the data sets (*p* < 0.05; Mann–Whitney U test).

The motility mutants shed significantly less LPS than the wild-type *V. fischeri* ES114 under both LSMMG and gravity conditions when normalized by optical density (Fig. 3). In wild-type cells, concentrations of reactionogenic LPS ranged between 5 × 10^4^ and 2.5 × 10^5^ EU per mL of media (Fig. 2a). In the motility mutants, however, the levels of reactogenic LPS were ~10-fold lower at each time point tested compared to the parent strain (Fig. 3a). By 14 h, however, there was a significant difference between the level of LPS measured in the LSMMG and gravity conditions in the motility mutants. Like the wild type, LSMMG-treated mutant cells exhibited significantly higher levels of LPS in both motility mutants compared to gravity controls (Fig. 3a). In addition, the *motB1* mutants shed significantly higher levels of LPS in the LSMMG conditions compared to the *flhA* mutant at 14, 16, and 20 h post inoculation (Fig. 3a). These differences were also independently confirmed with the Purpald assay (Fig. 3b) and suggest potential differences between the relative abundances between the components of the LPS molecule that are shed under LSMMG conditions.

The motility mutants displayed more modest growth increases compared to the parent strain, ES114 (Supplementary Fig. 1a–c) and to determine whether there were differences in cell size between the strains, cells collected from 12-h HARV cultures of wild type, *motB1*, and *flhA* mutants were stained with 1% crystal violet compare cell size under LSMMG and gravity treatments and observed with microscopy. Analyses of cell size revealed no significant difference between conditions or strains, suggesting optical density readings were directly comparable between LSMMG and gravity treatments for all strains (Supplementary Fig. 1d).

### Higher levels of LPS in LSMMG correlate with increased OMV production

To more fully understand the mechanics of increased LPS release under LSMMG conditions, the production of OMVs was examined under modeled microgravity. Almost all of the LPS released from *V. fischeri* cells has been shown to be associated with OMVs, which are produced largely due to flagellar rotation^35,36^. Therefore, we also examined whether OMV number and size were altered under the modeled microgravity conditions. Cultures of the wild type, *motB*1, and *flhA* strains were grown in LSMMG or gravity conditions and cell-free filtrates were evaluated at 12, 14, and 16 h post inoculation using nanoparticle tracking analysis. These times were chosen as they represent growth points with the largest discrepancies in LPS content between treatments observed across all three strains.

**Fig. 4:**
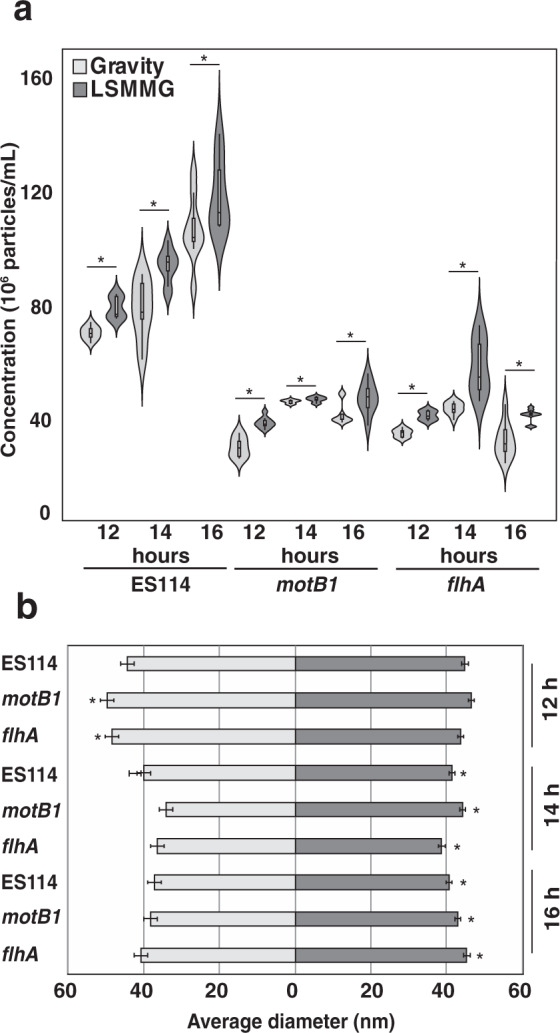
Nanoparticle size analysis of outer-membrane vesicles (OMVs) isolated from *Vibrio fischeri*. **a** Concentrations of OMVs in *V. fischeri* wild-type (ES114) and motility mutants (*motB1* and *flhA)* under gravity (light gray) and low-shear modeled microgravity (LSMMG, dark gray) conditions. **b** Average diameter of OMVs at 12, 14, and 16 h of incubation under gravity (light gray) and LSMMG (dark gray) conditions. Error bars indicate the standard error of the mean. Asterisks indicate significant differences between the data sets (*p* < 0.05; Mann–Whitney U test).

Nanoparticle analysis revealed a positive association between symbiont OMV vesiculation and modeled microgravity conditions. Specifically, the LSMMG filtrates of wild type, *flhA*, and *motB1* were found to contain significantly more OMV particles per mL than the gravity controls (Fig. 4a). Under both conditions, OMV concentration was observed to increase over time, with both *motB1* and *flhA* generating diminished vesicular yields relative to wild-type cells (Fig. 4a). In addition, by 14 h OMVs derived from LSMMG cultures were found to be significantly larger than gravity controls (Fig. 4b; Supplementary Table 1; Supplementary Dataset 1). At 16 h, the average size of OMVs for the modeled microgravity cultures was 40.72 ± 0.092 nm, whereas nanoparticle size averaged only 37.10 ± 0.092 nm for gravity controls (Supplementary Table 1). Similarly, in the *motB1* and *flhA* motility mutants the means of OMVs from LSMMG-treated cultures were 45.35 ± 0.068 and 43.05 ± 0.065 nm, respectively, whereas the mean size of gravity-treated OMVs was 40.72 ± 0.052 (*motB1*) and 38.16 ± 0.056 nm (*flhA*). Differences were also observed in the abundance of certain sized particles. Gravity samples tended to contain a higher proportion of smaller OMVs (diameter < 40 nm), whereas those cultivated in LSMMG exhibited a more even distribution (Supplementary Fig. 3). All isolated OMVs, whether they were derived from gravity or LSMMG conditions, exhibited a comparable ability to induce apoptosis and matched previously published levels of LPS and OMV-induced cell death (Supplementary Fig. 4)^21,35^.

### *V. fischeri* become increasingly sensitive to cell membrane-affecting agents in LSMMG

**Fig. 5:**
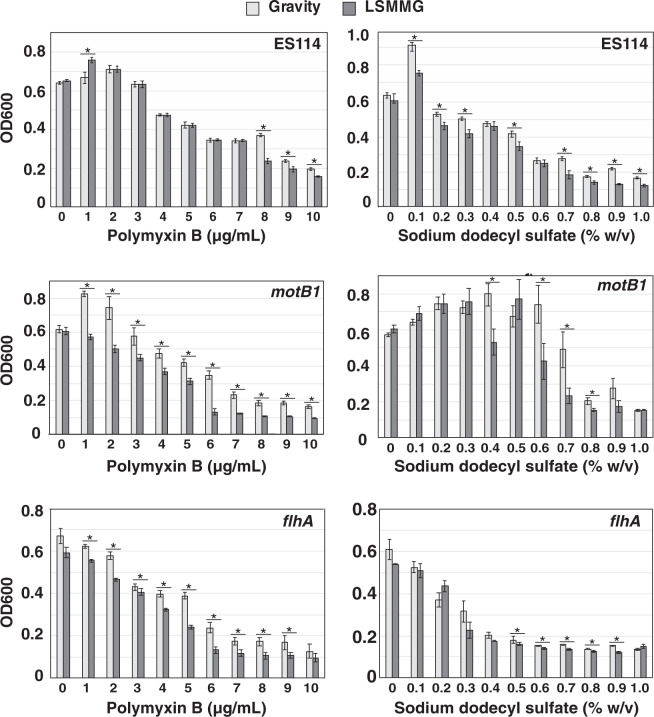
Dose–response of *Vibrio fischeri* cell densities in the presence of polymyxin B and sodium dodecyl sulfate. Cultures of wild-type *V. fischeri* ES114 and motility mutants (*motB1* and *flhA*) were grown for 12 h under gravity (light gray) and low-shear modeled microgravity (LSMMG) conditions. Error bars indicate the standard error of the mean. Asterisks indicate significant differences between the data sets (*p* < 0.05; Mann–Whitney U test).

**Fig. 6:**
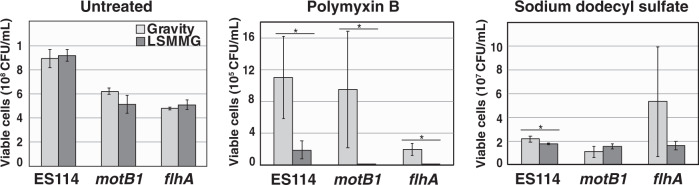
Direct cell plating counts for *Vibrio fischeri* exposed to polymyxin B and sodium dodecyl sulfate. Viable cell counts of wild-type *Vibrio fischeri* and motility mutants (*motB1* and *flhA*) in the presence of cell membrane-disruption agents polymyxin B and sodium dodecyl sulfate grown under gravity (light gray) and low-shear modeled microgravity (LSMMG, dark gray) conditions. Error bars indicate the standard error of the mean. Asterisks indicate significant differences between the data sets (*p* < 0.05; Welch’s *t*-test).

Although motility-dependent vesiculation is characteristic of species with sheathed flagella, such as *V. fischeri*^36^, all Gram-negative bacteria invariably produce OMVs by outer-membrane blebbing. OMV-blebbing is modulated by both external envelope stress and structural deficiencies in the bacterial cell wall^46^. Accordingly, the discovery that flagellar function does not, in fact, fully explain the model microgravity-associated differences observed in LPS-shedding or OMV release led us to hypothesize that LSMMG conditions may impact the integrity of the Gram-negative outer membrane. To investigate this, the susceptibility of *V. fischeri* toward a variety of cell membrane-affecting agents was compared in cells grown under LSMMG and gravity conditions, for the wild-type and motility mutants at 12, 14, and 16 h. Three specific agents were used to disrupt different components of the Gram-negative cell envelope. First, the cationic antibiotic polymyxin B was used, which binds to Gram-negative LPS and weakens the packing of lipid A molecules in the outer membrane^47^. Second, cells were exposed to the anionic detergent sodium dodecyl sulfate (SDS), which disrupts ionic interactions and causes bacterial cytoplasmic membrane lysis at high concentrations^48^. Last, cells were also exposed to a dilute solution of Triton X-100 for 1 min, which is a non-ionic detergent that disrupts lipid–lipid interactions^49^. After exposure, cell growth was assessed with optical density (Fig. 5; Supplementary Figs. 5 and 6) and plated to assess cell viability (Fig. 6).

Dose–response curves with 1–10 μg per mL polymyxin B and 0.1–1.0% v/v SDS revealed a concentration-dependent effect on bacterial growth in both LSMMG and gravity conditions. This effect was more pronounced in the LSMMG-treated strains at all time points tested (Fig. 5, Supplementary Figs. 5 and 6); LSMMG-treated *V. fischeri* generally showed increased sensitivity to membrane-disrupting agents. Despite showing different baseline sensitivities, the mutant strains also appeared more susceptible to the polymyxin B and SDS agents under LSMMG conditions compared to wild-type cells at all time points tested, with the polymyxin B treatment producing the most damaging effects to the motility mutants (Fig. 5, Supplementary Figs. 5 and 6). Plating of cells treated with polymyxin B- and SDS-treated cells revealed comparable results (Fig. 6) indicating that, regardless of strain, the viability of those cells exposed to polymyxin B were more negatively impacted when grown under LSMMG conditions.

**Fig. 7:**
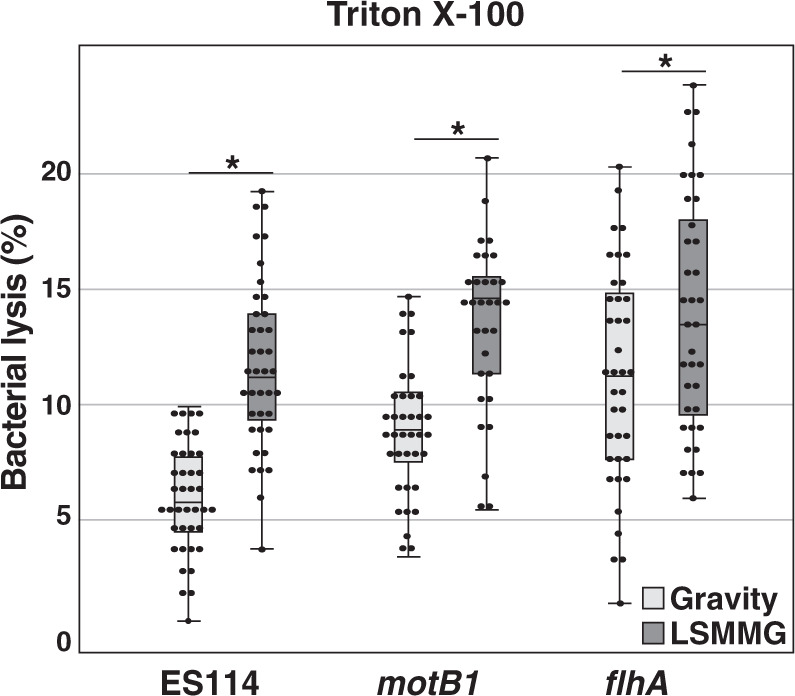
Percent lysis of *Vibrio fischeri* cultures following treatment with Triton X-100. The wild-type *V. fischeri* ES114 and motility mutants *motB* and *flhA* grown under gravity (light gray) and low-shear modeled microgravity conditions (LSMMG) for 12 h in response to 1 min of 0.05% v/v Triton X-100 exposure and then plated. Asterisks indicate significant differences between the data sets (*p* < 0.05; Mann–Whitney U test).

Similar results were also observed when cells were treated with Triton X-100. Cells of LSMMG-cultivated *V. fischeri*, regardless of strain, were also found to be more susceptible to the bactericidal effects of 0.05% Triton X-100, exhibiting more lysis than the gravity controls after 1 min of exposure (Fig. 7). To confirm that the results observed during these HARV experiments were not the product of changes to the background pH of the SWT media under LSMMG or gravity conditions, the pH was regularly monitored, and no significant changes to the pH were observed (Supplementary Fig. 7).

## Discussion

Over the coming decade, human-led exploration of space will likely move beyond low Earth orbit into deeper space^50^. Maintaining astronaut fitness during these long-duration space operations will require a detailed understanding of the microbiome under non-terrestrial conditions. To address this emerging issue in space biology, we used the HARV platform to model the effects of microgravity on *V. fischeri*, a beneficial microbe known to form symbiotic associations along animal epithelia. Results from this study indicated that (i) LSMMG conditions increased the shedding of bacterial LPS and associated OMVs into the surrounding media; (ii) the size of the OMV particles released during LSMMG were larger than gravity controls; (iii) flagellar motility in *V. fischeri* accounted for most of the overall OMV production, but was not necessary for increases in LPS shedding under LSMMG conditions; and (iv) the cell membranes of *V. fischeri* cells were more susceptible to disruption when cultivated under model microgravity conditions.

In Gram-negative bacteria, LPS is localized to the outer membrane and consists of a hydrophobic lipid A moiety, a core polysaccharide, and an O-antigen, or outward-projecting sugar chain of variable length^51,52^. LPS represents one of several MAMPs used by bacteria to either evade or communicate with their eukaryotic counterparts^53^. There has been an intense effort in recent years to understand how LPS is recognized by the host immune system through pattern recognition receptors^54^, and how this immune response is altered under microgravity conditions^55,56^.

The finding that symbiotic *V. fischeri* sheds more LPS under LSMMG conditions closely parallels previous studies that reported that microgravity analogs increases endotoxin load derived from the intestinal microbiota in rodents^55,56^. For example, in previous studies using the hindlimb unloading rodent model, a well-established method to simulate musculoskeletal disuse observed in spaceflight^57,58^, results indicated that both LPS and the associated host-derived lipopolysaccharide-binding protein were elevated in rodent serum under hindlimb unloading^56^. Levels of circulating LPS were further increased with concurrent exposure to proton or gamma radiation, another environmental hazard of spaceflight^55^. Although the increased levels in the serum were thought to be the result of breakdown of the gut epithelial barrier during hindlimb unloading, the root cause of the overall increased levels of LPS shedding by the microbiota into the host serum was not assessed^55,56^.

Interestingly, the results observed in *V. fischeri* differ from other host-associated taxa, such as *Salmonella enterica* serovar Typhimurium^17^. In previous studies, HARV-cultivated *Salmonella* exhibited significantly diminished levels of LPS O-antigen in LSMMG lysates, accompanied by a down-regulation of the *rfbP*, *rfbM*, and *rfbI* genes involved in O-antigen biosynthesis and ligation^17^. However, *V. fischeri* lacks homologs to these O-antigen synthesis genes^59^. In *V. fischeri* the O-antigen is comprised of a single pentasaccharide comprised of a two yersiniose, one 8-epi-legionaminic acid, and two n-acetyl-L-fucosamine residues^60^. The two quantitative techniques used in this study, LAL and Purpald assays do not directly measure the O-antigen so the differences between the two studies could reflect the methods used to quantify the LPS molecule as well as the different architectures of the LPS molecules. Alternatively, the differences in LPS release between the taxa under LSMMG conditions may simply reflect the fact that *Salmonella* does not have sheathed flagella, whereas *V. fischeri* has a membranous sheath that requires more torque to rotate, thereby releasing more LPS^61,62^.

In this study, the increased shedding of LPS from the bacterium *V. fischeri* under LSMMG conditions coincided with the exponential growth of the cultures (Fig. 2). In many taxa, including *V. fischeri*, higher cell densities and growth rates are observed under modeled microgravity conditions (Supplementary Fig. 1a, e)^63,64^. It has been proposed that the quiescent environment of LSMMG and continuous reorientation of the cell are altering the extracellular mass transfer of nutrients and waste, thereby impacting the cell’s growth rate^37,65^. Although *V. fischeri* is motile and there could be some mixing and disruption to the low-shear environment, *V. fischeri* may be accustomed to microgravity-like conditions as the symbionts regularly encounter low-shear environments when interfacing with the epithelial brush border of the host squid light organ tissues. Therefore, the observed increased growth rate may simply reflect the natural growth response for these symbiotic bacteria under low-shear stress environments.

The increase in LPS shedding under modeled microgravity conditions was also correlated to increases in the production of OMVs. The release of OMVs is an important mechanism by which bacteria interact with their environment^66^. OMVs derived from Gram-negative bacteria are produced in all stages of growth, and are frequently enriched with a range of bioreactive molecules, including LPS, outer-membrane proteins, and peptidoglycan-derivatives^46,67,68^. These discrete, enclosed structures are known mediators of host–microbe communication in both pathogenic and mutualistic associations^69^. In the squid-vibrio symbiosis, *V. fischeri* OMVs have previously been shown to induce hemocyte trafficking and apoptosis in the host symbiotic light organ, and are important mediators of bacteria-induced development in the host animal^43,68^. Both exogenous LPS and symbiont-derived OMVs can induce significant apoptosis in the light organ of uncolonized, aposymbiotic hatchlings, even at low concentrations (Supplementary Fig. 4). Thus, even small fluctuations in the amount of OMVs released in LSMMG could feasibly shift the timeline of symbiotic development.

The observed increase in the release of LPS containing OMVs during LSMMG conditions compared to gravity controls may suggest that the cells experience increased physiological stress in the modeled microgravity conditions, in particular, during exponential growth. Environmental stresses, such as temperature and osmotic stresses, can increase vesiculation in numerous Gram-negative bacteria^70^. The hypervesiculation of OMVs has been shown to relieve the cell envelope from waste products and the build-up of damaged, or misfolded, proteins, which accumulate during stress and normal growth^70,71^. Interestingly, the increased in OMV production under modeled microgravity conditions may also provide a possible mechanism for the increase resistance to environmental stresses and antibiotics observed in some microbes grown under both natural and microgravity conditions^37,72,73^. The ability of OMVs to quickly remove toxic material as well as absorb and bind antimicrobial agents may also serve as an important survival mechanism for microbes under stressful conditions, such as spaceflight.

One of the major mechanisms by which *V. fischeri* releases the OMVs is through flagellar rotation^36^. Certain members of the genus *Vibrio*, along with several other microbiome-associated taxa, such as *Helicobacter*, *Brucella*, and *Campylobacter*, produce flagella covered with a membrane-derived sheath^74–79^. These sheathed flagella have long been postulated to be a mechanism of communication between microbes and host tissues^43,69,80^. During rotation of the flagella, OMVs can then be released from both the tip and shaft of the flagella^81,82^. Mutants defective in flagella production, or in the rotation of the flagella, shed significantly fewer OMVs in comparison to wild-type strains^36^. This result is consistent with the *V. fischeri* strains grown in the HARV reactors, suggesting flagellar rotation is likely the primary, but not the only, mechanism by which OMVs are being released in both LSMMG and gravity conditions (Fig. 4). Interestingly, previous work found that flagella-driven increases in OMV release corresponded to smaller OMV size, whereas our results found that LSMMG-associated increases in OMV release corresponded with larger OMV sizes^36^. This result suggests that LSMMG presents a novel stress that stimulates a unique nonmotility- associated mechanism for OMV production changes in *V. fischeri*. In this vein, LSMMG stimulates increased production of OMVs across a range of bacteria with distinct lifestyles. Hypervesiculation has been reported in gentamycin-challenged spaceflight cultures of *E. coli*, a bacterium with unsheathed flagella, which unlike sheathed flagella, do not mediate LPS release^40^.

Our findings indicate the increased release of OMVs under modeled microgravity conditions may reflect changes in membrane integrity. Bacteria have previously been found to have altered cells membranes in both spaceflight and modeled microgravity conditions. For example, irregularities in cell volume, envelope integrity, and membrane polarization have been reported for *E. coli* and *Staphylococcus aureus* cultured in both natural and modeled microgravity conditions^63,83^. The increased susceptibility of *V. fischeri* strains to membrane-disruption agents (Figs. 5–7; Supplementary Figs. 5–6) further support the hypothesis that the cell membrane of *V. fischeri* is weakened by exposure to modeled microgravity conditions. In addition, previous analysis of the transcriptome of *V. fischeri* cells showed no differential expression of genes associated with LPS production during either exponential or stationary phase when grown under LSMMG^27^. Therefore, the results suggest that increases in the amount of LPS shed by *V. fischeri* occur primarily through mechanical means, and not through changes in LPS biosynthesis. Additional research is needed, however, to explore whether increased OMV shedding in LSMMG causes, or is caused by, LSMMG-related changes in cell membrane integrity.

Together, these results show that symbiotic microbes display significant physiological changes under modeled microgravity conditions, leading to a change in release of MAMPs known to affect host biology, which may have implications on host–microbe interactions. For example, in symbiotic bobtail squid exposed to modeled microgravity conditions the onset and peak of *V. fischeri*-induced apoptosis of the light organ’s superficial epithelium occurs significantly earlier than under gravity controls, which could be explained by increased OMV shedding^21^. Interestingly though, under LSMMG conditions there is also a delay in the trafficking of host immune cells within the superficial epithelium^21^, which is also typically triggered by MAMPs and is dependent on the presence of the outer-membrane protein OmpU in the OMVs^43,68^. This dissociated response in the host animal under LSMMG suggests microgravity may alter the content of the OMVs, or that the host may be responding differently to these bacterial-derived signals under microgravity, despite the lack of differences observed in the extent of LPS-induced cell death between LSMMG and gravity controls (Supplementary Fig. 4).

As many of the mechanisms that *V. fischeri* use to communicate with its host are shared amongst both pathogenic and beneficial microbes alike, our work provides important insight into how microgravity-like conditions may alter host–microbe relationships at the physiological level in general. Maintaining homeostasis of the microbiome during spaceflight is critical for the long-term host health, and these findings help build a critical framework to begin to explore the specific interactions and responses that occur between animals and their associated microbes.

## Methods

### Bacterial strains, media, and growth conditions

The strains of *V. fischeri* used in this study include: wild-type *V. fischeri* ES114^84^; the non-motile, flagellated, mutant *motB1*::Tn_7_*ermR;* and the non-flagellated mutant *flhA*::Tn_7_*ermR*^45^. For all experiments, *V. fischeri* was grown in seawater tryptone broth (SWT; 70% filtered ocean water containing 5 g L^−1^ tryptone, 3 g L^−1^ yeast extract, and 32.5 mM glycerol) at 28 ˚C with shaking. Where appropriate, media was supplemented with erythromycin to a final concentration of 5 μg per mL. Retention of the transposon insertions in the *motB1* and *flhA* motility mutants was verified for all experiments via soft-agar motility assays^85^. For assessments of growth, the concentration of *V. fischeri* was determined by reading the optical density at 600 nm, as an OD_600_ of 1.0 has been previously shown by plate counts to correspond with 3 × 10^8^ cells per mL of culture^84,86^ and was independently confirmed in the HARVs. To ensure that background levels of OMVs did not significantly alter the OD_600_ readings, 1 mL of *V. fischeri* culture was filtered to remove bacteria using a 0.22 µm syringe filter and measured spectrophotometrically. No significant (*p* < 0.0001) difference in optical density was observed in the spent media from background SWT media.

### Electron microscopy

All scanning electron micrographs (SEM) and transmission electron micrographs (TEM) were acquired at the University of Hawai’i Biological Electron Microscopy Facility. For SEM, *V. fischeri* were grown in SWT media to mid-log phase (OD_600_ 0.4–0.6) with shaking at 28 °C. For TEM, cells were grown under similar conditions in LB salt (LBS) media containing 10 g L^−1^ tryptone, 5 g L^−1^ yeast extract, 342 mM NaCl, and 20 mM Tris at pH 7.5.

Samples visualized with SEM were fixed with marine fixative containing 2.5% glutaraldehyde, 2% paraformaldehyde, 350 mM sucrose, 100 mM sodium cacodylate at pH 7.6 for 1 h. The cells were then washed in 100 mM sodium cacodylate buffer containing 400 mM sucrose and post-fixed with 100 mM sodium cacodylate containing 1% osmium tetraoxide for 1 h. After fixation the cells were collected on a 0.22-μm filter, then serially dehydrated in ethanol and dried with a Tousimis Samdri-795 critical point dryer. Filters were mounted on aluminum stubs and sputter coated with a Hummer 6.2 sputter coater, then imaged with a Hitachi S-4800 Field Emission Scanning Electron Microscope with accelerating voltage.

TEM was performed as previously described^68^. Briefly, *V. fischeri* cells were fixed, washed, and post-fixed similarly to the samples prepared for SEM. Fixed *V. fischeri* cells were then serially dehydrated in a graded ethanol series, infiltrated with propylene oxide, then embedded and polymerized in LX112 epoxy resin for 2 days. Ultrathin sections were stained with a saturated solution of lead citrate and uranyl acetate and imaged with a Hitachi HT7700 transmission electron microscope coupled to an AMT XR-41B 2k x 2k CCD camera.

### Low-shear modeled microgravity conditions

To model the LSMMG environment, bacterial cultures were inoculated to a final concentration of 1 × 10^5^ cells per mL of SWT and then loaded into 50-mL capacity HARVs (Synthecon, Houston, TX), as previously described^21,27^. HARVs were incubated at 23 °C and rotated at 13 rpm to mimic spaceflight temperatures and to prevent sedimentation of the cultures. Gravity controls were rotated around a vertical axis to control for HARV-specific effects. To assess growth, the OD_600_ of the HARV cultures were measured in triplicate every 2 h using a Synergy plate reader (BioTek, Winooski, VT). To examine the effects of LSMMG on *V. fischeri* size, cultures from each HARV were collected at 12 h. Cells were stained with 1% crystal violet and analyzed with a Zeiss Axioplan microscope, using PROGRES GRYPHAX Image Capture software for measurements of cell length (Carl Zeiss, Jena, Germany). To monitor motility of the LSMMG- and gravity-treated cells, aliquots were also examined with both direct light microscopy video imaging using PROGRES GRYPHAX Image Capture software and soft-agar motility assays as previously described^85^. All experiments were performed at least three times and outliers identified via the 1.5× interquartile range (IQR) method were removed prior to analysis^87^. A Shapiro–Wilk test was used to assess data normality. Significance was determined via non-parametric Mann–Whitney U test using a cut off of *p* < 0.05.

### LPS preparation, treatments, and quantification

For the quantification of *V. fischeri*-derived LPS, cells were spun down by centrifugation and the resulting supernatant was sterilized by successive passage through 0.45- and 0.22-μm syringe filters. Reactionogenic LPS within the cell-free filtrates was then examined using the ToxinSensor^TM^ Chromogenic Limulus Amebocyte Lysate (LAL) Endotoxin Assay Kit (Genscript, Piscataway, NJ) according to the manufacturer’s instructions. For all samples, the absorbance at 545 nm was used to calculate the concentration of reactionogenic LPS in endotoxin units (EU) per mL, which was subsequently normalized to the OD_600_ of the culture to account for differences in growth between LSMMG and gravity conditions within the HARVs. For all LAL assays, 0.10, 0.25, 0.50, and 1.0 EU per mL standards were run in triplicate.

In addition, total LPS was quantified using the colorimetric Purpald assay^41^ with a Synergy plate reader (Biotek, Winsooki, VT). The Purpald assay is based on the sequential periodate oxidation of LPS core-specific sugar residues and the reaction of formaldehyde with Purpald Reagent (4-amino-3-hydrazino-5-mercapto-1,2,4,triazole) to produce a purple-colored end product^41^. For all samples, the absorbance at 550 nm was used to calculate the concentration of total LPS in μg per mL, which was normalized by OD_600_ to account for differences in growth. The test:control ratio comparing total LPS from cultures grown in LSMMG versus normal gravity conditions was then calculated. For all Purpald assays, 10, 25, 50, 100, 250, and 500 μg per mL LPS standards were run in triplicate. Outliers identified via the 1.5× interquartile range (IQR) method were removed prior to analysis and a Shapiro–Wilk test was used to assess data normality. Significance was determined via non-parametric Mann–Whitney U test using a cut off of *p* < 0.05.

### Isolation and quantification of OMVs from *V. fischeri* ES114

To isolate OMVs, 1 L of *V. fischeri* cultures were grown under LSMMG and gravity conditions in the HARVs. Cells were pelleted by centrifugation, and the resulting supernatant passed through 0.45 and 0.22-μm filters (Millipore Sigma, Burlington, MA) to yield a purified, cell-free, filtrate. On those OMV samples exposed to host animals, the vesicles were pelleted by ultra-centrifugation (TL-100, Beckman, Brea, CA) at 173,000 × *g* for 2 h at 4 °C to concentration the OMV particles. The pellet was then washed and resuspended in phosphate-buffered saline (PBS) supplemented with 0.20 M NaCl to yield a crude OMV extract. The OMVs in this extract were further separated from cellular debris by additional ultra-centrifugation at 90,000 × *g* for 15 h at 4 °C through a discontinuous 25–55% w/v sucrose density gradient. After, the fraction upward of 45% was collected and washed twice with PBS by ultra-centrifugation at 173,000 × *g* for 4 h at 4 **˚**C. The final OMV preparation was filter-sterilized through a 0.22-μm filter. OMV yield was measured by protein assay using a Qubit 2.0 fluorometer (Life Technologies, Carlsbad, CA) according to the manufacturer’s instructions. All final OMV preparations were stored at −80 °C until use.

The concentration and size distributions of OMVs produced under LSMMG and gravity conditions were determined by nanoparticle tracking analysis using a NanoSight NS300 instrument equipped with a 488 nm laser (NanoSight, Ltd., Salisbury, UK). Cell-free filtrates from wild-type and motility mutants at 12, 14, and 16 h were diluted in PBS to achieve a threshold of 10–100 particles per frame. Prior to analysis, background values were subtracted from all measurements of concentration. All measurements were taken at ambient temperature (22–24 °C), with the settings for minimum expected particle size, shortest track length, and blur left at the factory default. For each sample, a minimum of 40-s capture videos was collected in triplicate, with the camera operating at the standard 30 frames-per-second. Between samples, the NS300 system was flushed thrice with PBS and once with deionized water. Further analyses to determine mean particle size, size distribution, and concentration were performed using the Nanoparticle Tracking Analysis software suite (NTA; version 3.2, NanoSight, Ltd., Salisbury, UK). Background particle concentration was assessed by diluting and analyzing sterile SWT. A Shapiro–Wilk test was used to assess the normality of the nanoparticle concentration data. The normality of the OMV size data—which contained over 10,000 measurements per condition—was determined with a Lillifors test^88^. Significance was determined via non-parametric Mann–Whitney U test using a cut off of *p* < 0.05.

### General *E. scolopes* procedures and visualization of host apoptotic cells

Squid experiments were conducted in triplicate and in accordance with the University of Florida and Kennedy Space Center Institutional Animal Care and Use Committees. After hatching, juvenile squid was rinsed twice in 0.22 μm filtered seawater (FSW) and treated with either exogenous LPS from *Salmonella enterica* serovar Enteritidis (Sigma Aldrich, St. Louis, MO) or OMVs isolated from batch cultures of the wild-type *V. fischeri*. A subset of hatchlings was also rendered symbiotic by inoculation with the wild-type *V. fischeri* to a final concentration of 1 × 10^5^ cells per mL of FSW. After 16 h, the animals were anesthetized in a 1:1 solution of FSW and 0.37 MgCl_2_ for 5 min, containing 0.001% acridine orange dye. Light organ apoptosis was then examined via epifluorescence microscopy using a Zeiss Axioplan Microscope (Carl Zeiss, Jena, Germany). Colonization was verified for all treatments by measuring bacterial luminescence with a photometer (GloMax 20/20 Luminometer, Promega Corp., Madison, WI).

### Membrane integrity evaluation under gravity and LSMMG conditions

To examine the effects of LSMMG on *V. fischeri* cell wall integrity, growth curves were conducted in triplicate in the presence of polymyxin B and SDS. Samples were collected from each HARV, normalized, and exposed to a solution containing either 1–10 μg polymyxin B or 0.1–1.0% SDS per mL of SWT at 23 °C without shaking. After 24 h of exposure, the samples were resuspended by pipetting and optical density was measured spectrophotometrically (OD_600_) and significance was determined via non-parametric Mann–Whitney U test for all readings of optical density. To measure the viability of cells treated with the polymyxin B or SDS a subset of cells was then diluted and plated on SWT media. Significance between treatments of plate counts was assessed using a Welch’s *t*-test with a cut off of *p* > 0.05.

To further assess the integrity of the symbiont cell membrane under LSMMG conditions, cultures were grown for 12 h at 23 °C and pelleted in 5 mL aliquots. The pellets were washed and resuspended in PBS and then incubated in 0.05% v/v Triton X-100, a non-ionic detergent that disrupts membrane lipids^48^ at 23 °C for 1 min. Immediately after, the absorbance was read at 405 nm and the percentage of intact, undisrupted, cells was calculated as the turbidity of the treatment group relative to the control. This value was then subtracted from 100% to obtain the percent lysis. The formula is as follows: Bacteriolysis (%) = (100% − ((OD_405_ of treatment/OD_405_ of control) × 100)). A Shapiro–Wilk test was used to assess data normality whereas significance was determined via non-parametric Mann–Whitney U test.

### Reporting summary

Further information on research design is available in the Nature Research Reporting Summary linked to this article.

## Supplementary information

Supplementary Information

Supplementary Data 1

Reporting Summary Checklist

## Data Availability

All data generated or analyzed during this study are included in this published article and its Supplementary information files.
