# Supplementary material for: Modeled microgravity alters lipopolysaccharide and outer membrane vesicle production of the beneficial symbiont Vibrio fischeri
Source: NPJ Microgravity. 2021 Mar 8;7:8. doi: 10.1038/s41526-021-00138-8 (PMC7940393; doi:10.1038/s41526-021-00138-8)
Supplement: Supplementary file 1 — Supplementary Information [file 41526_2021_138_MOESM1_ESM.pdf]

Supplementary Information

Modeled microgravity alters lipopolysaccharide and outer membrane vesicle production of the beneficial symbiont *Vibrio fischeri*

Madeline M. Vroom[1], Yaneli Rodriguez-Ocasio[1], Jonathan B. Lynch[2*], Edward G. Ruby[2], and Jamie S. Foster[1**]

Running title: Modeled microgravity impacts beneficial symbiont

[1]Department of Microbiology and Cell Science, Space Life Science Lab, University of Florida, Merritt Island, FL 32953, USA.

[2]Pacific Biosciences Research Center, Kewalo Marine Laboratory, University of Hawai‘i at Manoa, Honolulu, HI, 96813, USA.

*Department of Integrative Biology and Physiology, University of California, Los Angeles, CA 90095, USA

**Corresponding Author:

jfoster@ufl.edu; 321-525-1047

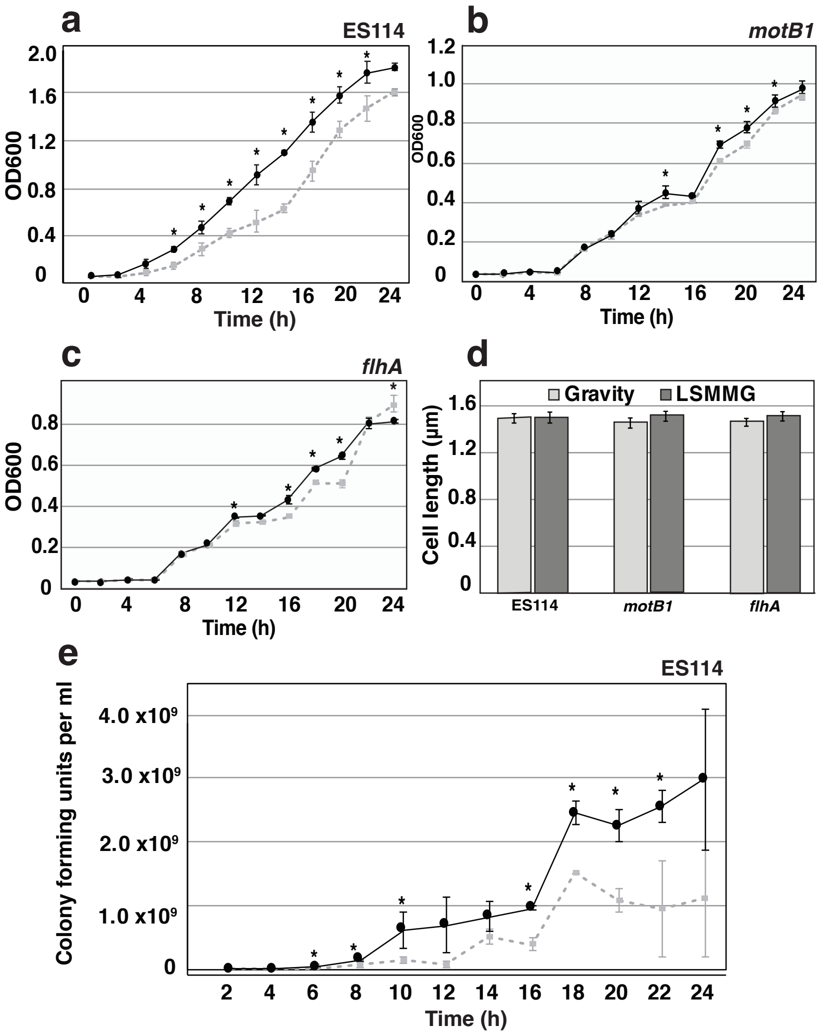

Supplementary Fig. 1 Growth curves of *Vibrio fischeri* strains grown in the rotating wall cell culture system. Strains included: **a** *Vibrio fischeri* ES114 wild-type, motility mutants; **b** *motB1*; and **c** *flhA* grown under both gravity (dotted line) and low shear modeled microgravity (LSMMG, solid line) conditions. **d** Comparison of cell length of *V. fischeri* strains under gravity (light gray) and LSMMG (dark gray) conditions. **e** Growth curves of wild-type ES114 as determined by direct cell plating on SWT media under gravity (dotted line) and LSMMG (solid line) conditions. Error bars indicate the standard error of the mean. Asterisks indicate significant differences between the data sets ($p < 0.05$; Mann-Whitney U test).

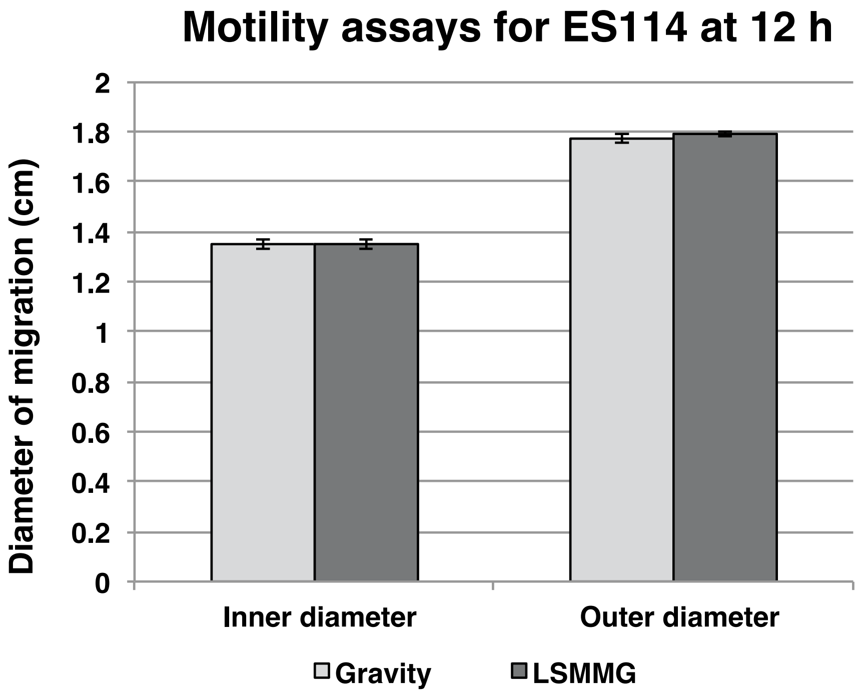

Supplementary Fig. 2 Motility assay results for wild-type *Vibrio fischeri* in 0.3% sea water trypone soft agar following 12 h cultivation in gravity versus low shear modeled microgravity (LSMMG) conditions. Error bars indicate the standard error of the mean. Asterisks indicate significant differences between the data sets ($p < 0.05$; Mann-Whitney U test).

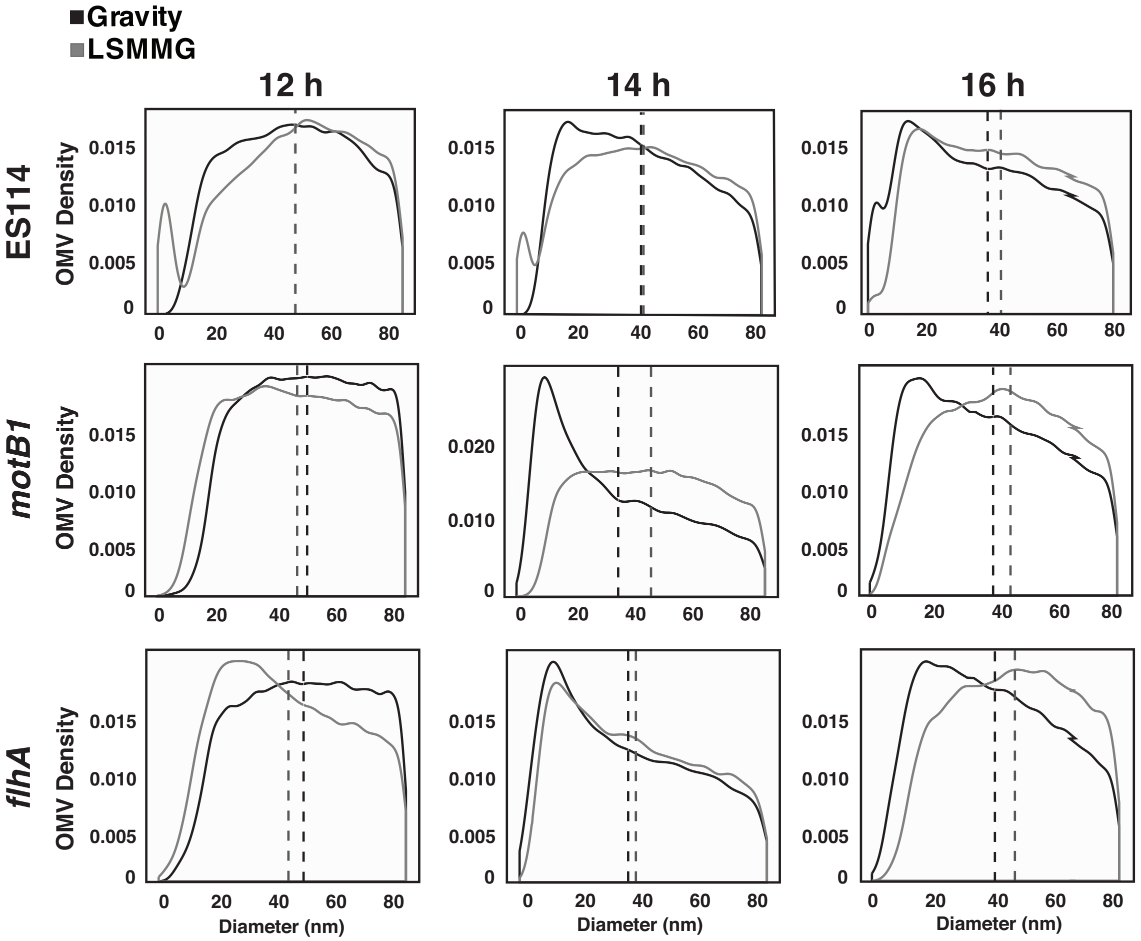

Supplementary Fig. 3 Density plots illustrating the average size and overall distribution of putative outer membrane vesicles isolated from wild-type *Vibrio fischeri* ES114 and motility mutants *motB1* and *flhA* shed under gravity and low shear modeled microgravity (LSMMG) conditions.

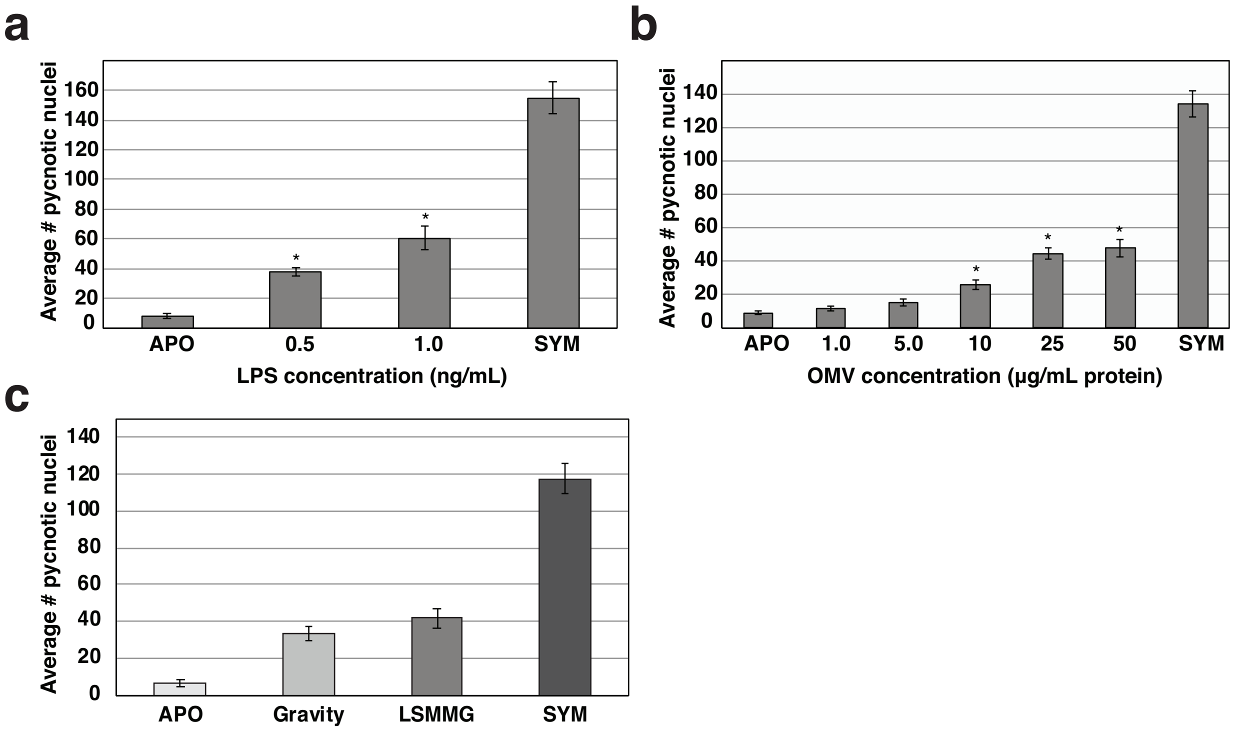

Supplementary Fig. 4 Validation of outer membrane vesicle (OMV) preparations in the onset of pycnotic cell death, a hallmark of apoptosis, in host animal at 16 h. **a** Control exogenous LPS induced a dose-dependent curve of apoptotic cell death in aposymbiotic (APO) host light organ superficial epithelium in comparison to *Vibrio fischeri* (SYM)-treated animals response. **b** Purified OMVs from cells grown under normal gravity conditions induce a similar dose-dependent increase in the numbers of pycnotic nuclei in host light organ validating the isolation protocol used in this study. Error bars indicate the standard error of the mean. **c** Normalized OMVs purified from gravity and LSMMG conditions induced comparable levels of apoptotic cell death in the host animal. Both populations of OMVs were higher than aposymbiotic (APO) controls, but lower than normal cell death levels observed in animals colonized by intact *V. fischeri* (SYM). Asterisks indicate significant differences between the data sets ($p < 0.05$; Welch's t-test).

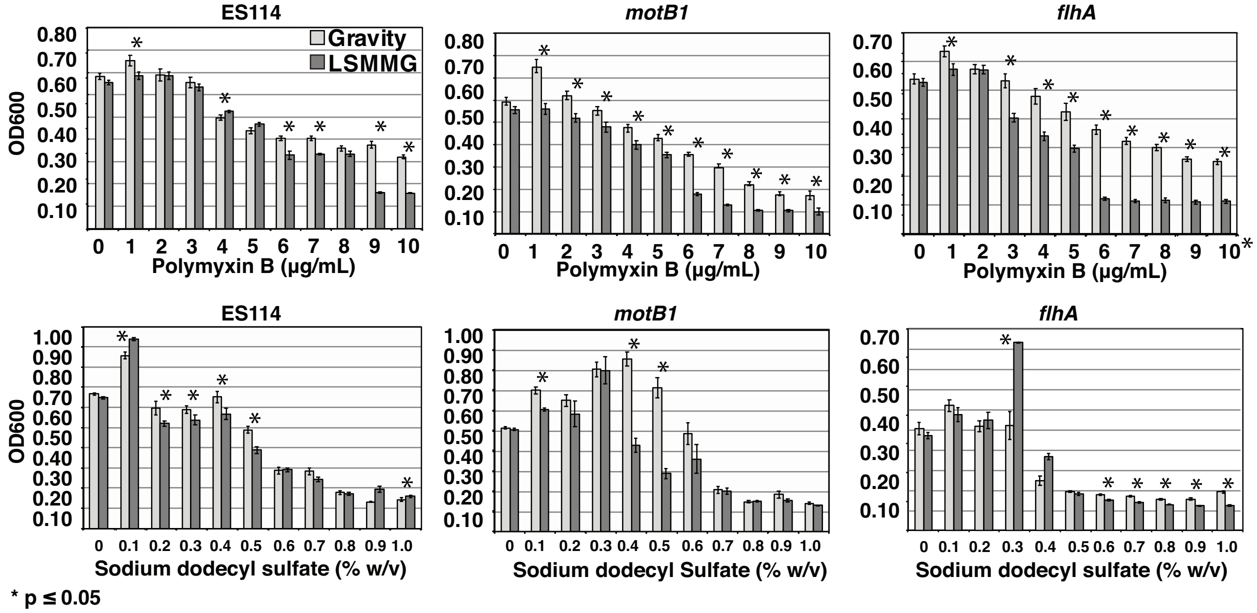

Supplementary Fig. 5 Dose-response responses of *Vibrio fischeri* cell densities in the presence of cell membrane disruption agents polymyxin B and sodium dodecyl sulfate. Cultures of wild-type *V. fischeri* ES114 and motility mutants (*motB1* and *flhA*) were grown for 14 h under gravity (light gray) and low shear modeled microgravity (LSMMG) conditions the presence of the agents. Error bars indicate the standard error of the mean. Asterisks indicate significant differences between the data sets ($p < 0.05$; Mann-Whitney U test).

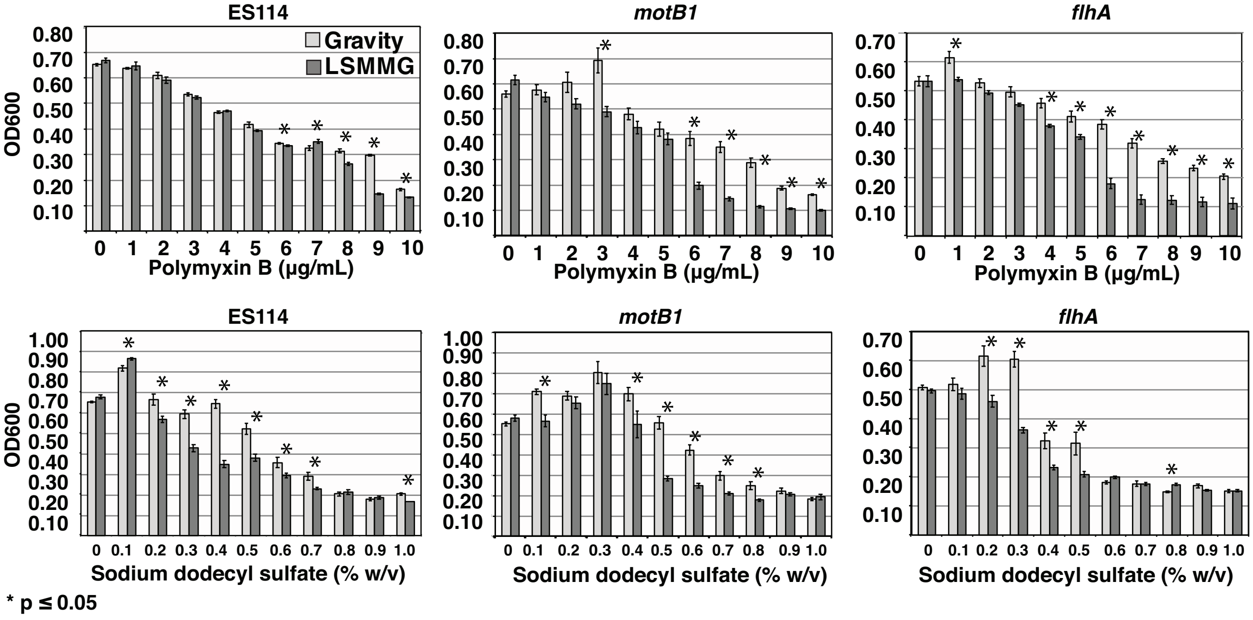

Supplementary Fig. 6 Dose-response responses of *Vibrio fischeri* cell densities in the presence of cell membrane disruption agents polymyxin B and sodium dodecyl sulfate. Cultures of wild-type *V. fischeri* ES114 and motility mutants (*motB1* and *flhA*) were grown for 16 h under gravity (light gray) and low shear modeled microgravity (LSMMG) conditions in the presence of the agents. Error bars indicate the standard error of the mean. Asterisks indicate significant differences between the data sets ($p < 0.05$; Mann-Whitney U test).

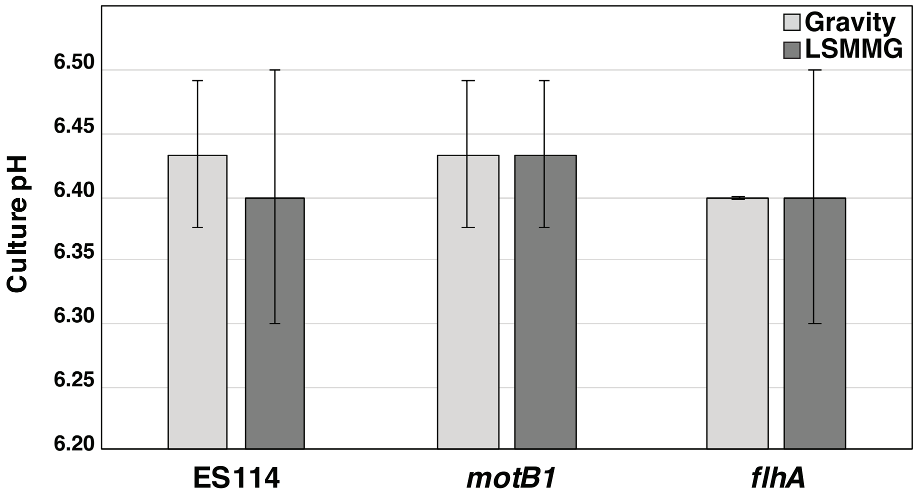

Supplementary Fig. 7 Measurements of pH of the surrounding media of wild-type *Vibrio fischeri* ES114 and motility mutants *motB1* and *flhA* cultivars grown for 12 h within the high aspect ratio vessels under both gravity and low shear modeled microgravity (LSMMG) conditions. Error bars represent the standard error of the mean. Error bars indicate the standard error of the mean. Asterisks indicate significant differences between the data sets ($p < 0.05$; Mann-Whitney U test).

Supplementary Table 1. Mean size of outer membrane vesicles from gravity and low shear modeled microgravity (LSMMG) over time as determined by nanoparticle size analysis.

Strain	Time (h)	Gravity-treated (nm)[a]	LSMMG-treated (nm)[a]
ES114	12	44.33 ± 0.092	44.86 ± 0.092
motB1	12	49.64 ± 0.056	46.54 ± 0.065
flhA	12	48.44 ± 0.052	43.78 ± 0.068
ES114	14	40.02 ± 0.094	41.42 ± 0.085
motB1	14	34.01 ± 0.094	44.30 ± 0.075
flhA	14	36.32 ± 0.111	38.74 ± 0.098
ES114	16	37.10 ± 0.100	40.72 ± 0.089
motB1	16	38.16 ± 0.090	43.05 ± 0.082
flhA	16	38.84 ± 0.081	45.35 ± 0.079

[a] Values correspond to Fig. 4b.

Supplementary Dataset 1. Separate dataset available with the raw Nanosight measurements (nm) of putative outer membrane vesicle diameters at 16 hours.
